# Statin use after intracerebral hemorrhage: a 10‐year nationwide cohort study

**DOI:** 10.1002/brb3.487

**Published:** 2016-05-13

**Authors:** Shu‐Yu Tai, Feng‐Cheng Lin, Chung‐Yin Lee, Chai‐Jan Chang, Ming‐Tsang Wu, Chen‐Yu Chien

**Affiliations:** ^1^ Department of Family MedicineSchool of MedicineCollege of MedicineKaohsiung Medical UniversityKaohsiungTaiwan; ^2^ Department of Family MedicineKaohsiung Medical University HospitalKaohsiung Medical UniversityKaohsiungTaiwan; ^3^ Department of Family MedicineKaohsiung Municipal Ta‐Tung HospitalKaohsiung Medical University HospitalKaohsiung Medical UniversityKaohsiungTaiwan; ^4^ Department of NeurologyKaohsiung Medical University HospitalKaohsiungTaiwan; ^5^ Department of NeurologyPingtung HospitalMinistry of Health and WelfarePingtungTaiwan; ^6^ Department of Family MedicineKaohsiung Municipal Hsiao‐Kang HospitalKaohsiung Medical UniversityKaohsiungTaiwan; ^7^ Department of Public HealthKaohsiung Medical UniversityKaohsiungTaiwan; ^8^ Center of Environmental and Occupational MedicineKaohsiung Municipal Hsiao‐Kang HospitalKaohsiung Medical UniversityKaohsiungTaiwan; ^9^ Department of OtorhinolaryngologySchool of MedicineCollege of MedicineKaohsiung Medical UniversityKaohsiungTaiwan; ^10^ Department of OtorhinolaryngologyKaohsiung Medical University HospitalKaohsiung Medical UniversityKaohsiungTaiwan; ^11^ Department of OtorhinolaryngologyKaohsiung Municipal Hsiao‐Kang HospitalKaohsiung Medical UniversityKaohsiungTaiwan

**Keywords:** Intensity, intracerebral hemorrhage, solubility, statin

## Abstract

**Introduction:**

Although statin therapy is beneficial to patients with ischemic stroke, statin use, and intracerebral hemorrhage (ICH) remain a concern. ICH survivors commonly have comorbid cardiovascular risk factors that would otherwise warrant cholesterol‐lowering medication, thus emphasizing the importance of assessing the characteristics of statin therapy in this population.

**Methods:**

We performed a cohort study by using 10 years of data collected from the National Health Insurance Research Database in Taiwan. We enrolled 726 patients admitted for newly diagnosed ICH from January 1, 2001 to December 31, 2010. The patients were categorized into high‐ (92), moderate‐ (545), and low‐intensity (89) statin groups, and into hydrophilic (295) and lipophilic (431) statin groups. The composite outcomes included all‐cause mortality, recurrent ICH, ischemic stroke, transient ischemic attack, and acute coronary events.

**Results:**

The patients in the low‐intensity group did not differ significantly from the patients in the high‐intensity group in risk of all‐cause mortality (adjusted hazard ratio [aHR] = 0.65, 95% confidence interval [CI] = 0.28–1.55) and recurrent ICH (aHR = 0.66, 95% CI = 0.30–1.44). In contrast, the patients in the hydrophilic group had a significantly lower risk of recurrent ICH than did those in the lipophilic group (aHR = 0.69, 95% CI = 0.48–0.99). We determined no significant differences in other composite endpoints between hydrophilic and lipophilic statin use.

**Conclusion:**

Hydrophilic statin therapy is associated with a reduced risk of recurrent ICH in post‐ICH patients. The intensity of statin use had no significant effect on recurrent ICH or other components of the composite outcome. Additional studies are required to clarify the biological mechanisms underlying these observations.

## Introduction

Intracerebral hemorrhage (ICH) causes approximately 15% of all strokes in Western populations and 20–30% of all strokes in the Chinese population (Zhao et al. 2008; Wang et al. 2011). Patients with ICH generally have a poorer prognosis and higher mortality than do those with ischemic stroke (Biffi et al. 2011). Currently, no proven effective treatment for ICH exists. Therefore, preventing ICH to reduce the healthcare burden associated with hemorrhage stroke and other corresponding negative outcomes is critical.

HMG‐CoA reductase inhibitors, commonly known as statins, have been demonstrated to be beneficial in the primary and secondary prevention of ischemic stroke (Amarenco and Labreuche 2009; Stone et al. 2013). However, concerns have been raised about the risk of cerebral hemorrhage associated with statin use (Mascitelli and Pezzetta 2009; Vergouwen et al. 2009). This risk might be of major relevance to patients with prior ICH, who are at high risk of hemorrhage, which could result from the statin‐induced reductions in cholesterol levels and inhibition of platelet aggregation (Ricard et al. 2010). These concerns are particularly relevant to Asians, a population at higher risk of ICH (Tanaka et al. 1982; Hu et al. 1992).

Animal studies have shown that statins benefit neurological recovery, endothelial stabilization, and anti‐inflammatory activity because of their ability to reduce lipids (Giannopoulos et al. 2012; Yang et al. 2013a,b). However, statins may also have antithrombotic properties because they can inhibit platelet aggregation and enhance fibrinolysis, which might amplify the risk of ICH (Goldstein 2013). Most studies have supported the use of statins in patients before ICH (Leker et al. 2009; Eichel et al. 2010; Gomis et al. 2010; King et al. 2012; Mustanoja et al. 2013; Winkler et al. 2013) and after ICH (Flint et al. 2014; Pan et al. 2014; Chen et al. 2015), and they have concluded that statins promote favorable functional outcome and low mortality. However, most of those studies have focused on the association between the timing of statin use (Leker et al. 2009; Eichel et al. 2010; Gomis et al. 2010; King et al. 2012; Mustanoja et al. 2013; Winkler et al. 2013; Flint et al. 2014; Pan et al. 2014; Chen et al. 2015) or continuing (discontinuing) statin therapy (Dowlatshahi et al. 2012; Bustamante and Montaner 2013; Goldstein 2013; Scheitz et al. 2013; Tapia‐Perez et al. 2013) and the outcomes after a diagnosis of ICH. They have rarely considered the differences in the types of statin used or the differences in regimens and their association with outcomes in patients with newly diagnosed ICH. In addition, the mean follow‐up duration in statin and ICH trials is short, usually lasting 24 months or fewer. We performed a 10‐year population‐based cohort study to explore the relationship between the characteristics of post‐ICH statin use and the long‐term outcomes in patients with newly diagnosed ICH. The findings of such a study can provide useful information on the effective clinical use of these agents in patients after diagnosis of ICH for reducing the risk of recurrent ICH and hemorrhage stroke.

## Methods

### Ethics statement

The database we used comprised de‐identified secondary data, and the study met the requirements of the Personal Information Protection Act of Taiwan. Because the analyzed data were anonymized, the need for informed consent was waived by the Institutional Review Board of Kaohsiung Medical University Hospital, Taiwan (KMUHIRB‐EXEMPT (I)‐20150027).

### Data sources

We conducted this cohort study by using a sampling cohort data set obtained from the National Health Insurance Research Database (NHIRD) containing claims data for patients enrolled in Taiwan's National Health Insurance (NHI) program. The program has provided mandatory universal health insurance since 1995, covers up to 99% of all the residents of Taiwan, and contracts with 97% of all the medical providers in Taiwan. The NHIRD is a randomly selected data set of one million NHI claims from the year 2000 registry of all NHI enrollees (NHI 2000). We retrospectively and prospectively followed these patients from January 1, 1997 to December 31, 2010. According to the National Health Research Institutes, there are no significant differences in age, sex, and healthcare costs between the sampled group and all enrollees in NHI 2000 (National Health Insurance Research Database 2000). The database contains comprehensive demographic data, including sex, date of birth, and income level, as well as healthcare data, including the date of admission or discharge, clinical diagnoses (up to five coexisting diagnoses listed on one claims record), medical procedures (up to five diagnostic or therapeutics procedures), expenditures, detailed drug prescriptions, and in‐hospital deaths. The NHI lists diagnoses according to the codes of the *International Classification of Diseases, Ninth Revision, Clinical Modification* (ICD‐9‐CM) (2005).

### Cohort population

We enrolled participants admitted for newly diagnosed ICH from January 1, 2001 to December 31, 2010 who had received initial brain computed tomography (CT) scans upon admission. We screened eligible participants on the basis of the principal diagnosis recorded in the NHIRD. We identified 4896 patients with a diagnosis of ICH (ICD‐9 CM: 431). Participants were excluded if they (1) had been admitted to hospitals because of a recurrent ICH within 1 year of their recruitment (*n *=* *97), (2) had not used statin after the ICH event for which they were recruited (*n *=* *3992), (3) had incomplete registration information (*n *=* *23), (4) had incomplete medical records within 1 year of their recruitment (*n *=* *37), or (5) had no available follow‐up data after discharge (*n *=* *21). We finally enrolled 726 patients for the statistical analysis.

### Definition of the characteristics of statin initiation

**Figure 1:**
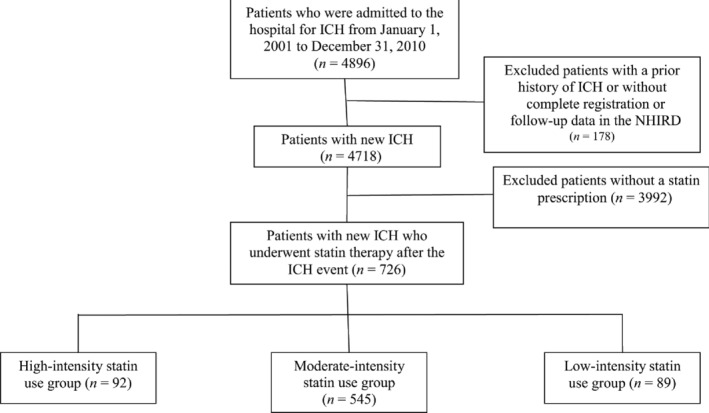
Participant enrollment flowchart.

Patients with at least one statin prescription after enrollment were studied, and their first prescription of statin was defined as their initial usage. We categorized initial statin usage into high‐, moderate‐, and low‐intensity categories according to the average expected LDL–C response to a specific statin and dose outlined in the *2013 ACC/AHA Guideline* (Stone et al. 2013). Of the 726 patients, 92 were categorized as “high‐intensity” statin users, 545 as “moderate‐intensity” statin users, and 89 as “low‐intensity” statin users. Additional analyses were performed, and they entailed stratifying the statin therapies by hydrophilic and lipophilic solubility (water and oil solubility) (Serajuddin et al. 1991; Rosenson 2004). Moreover, of the enrolled patients, 431 were categorized as lipophilic statin users and 295 as hydrophilic statin users. Figure 1 displays a flowchart of participant enrollment and category assignment.

### Study endpoints and follow‐up

The primary outcome of interest was recurrent ICH (ICD‐9‐CM code 431) diagnosed through CT scanning during admission. Secondary endpoints included individual components of ischemic stroke (ICD‐9‐CM codes 433, 434, 436, 437.1, and 437.9), transient ischemic attack (TIA; ICD‐9‐CM code 435), or acute coronary events (ICD‐9‐CM code 410). Moreover, all‐cause mortality was included as of one these secondary endpoints. However, because some Taiwanese patients prefer to pass away at home rather than in a hospital, the NHIRD contains no death records for patients who passed away at home. To determine deaths, we first assumed that patients with ICH required long‐term follow‐up and medication use; we subsequently assumed that a patient had passed away when he or she had no claims data associated with a physician visit or prescription in the NHIRD for more than 30 days following ICH diagnosis or when he or she had a death record in the NHIRD. The occurrence of other negative study outcomes (recurrent ICH, acute coronary events, or ischemic stroke or attack) was identified on the basis of diagnoses recorded in the ambulatory and hospitalization claims of the NHIRD. Eligible patients were followed up from the date of their first statin prescription to the date of their final medical record or before the end of the study period (December 31, 2010). We censored patients who did not reach the outcomes of interest.

### Statistical analysis

The basic characteristics, comorbid diseases, and medical histories of each participant were recorded and analyzed from the data registered in the NHIRD. Charlson comorbidity index adjustments to the ICH outcomes were used to adjust the comorbidity of each participant (Goldstein et al. 2004). Continuous variables are described as means ± standard deviation, and categorical variables are described in patient numbers and percentages. ANOVA or a Student's *t* test was used for normally distributed continuous variables between groups, whereas chi‐squared tests were used to compare categorical variables. Variables with a two‐tailed *P* value <0.05 were considered significant. The event‐free time to the composite endpoints is presented as a Kaplan–Meier survival curve stratified by the characteristics of statin initiation. The time‐dependent Cox proportional hazards model was used to examine the relationship between the endpoints and characteristics of initial statin therapy. Hazard ratios (HRs) and their corresponding 95% confidence intervals (CIs) were calculated. All statistical operations were performed using SAS for Windows Version 9.2 (SAS Institute, Inc., Cary, NC).

## Results

### Baseline characteristics of different groups

Tables 1 and 2 show the baseline characteristics of the different groups of patients with newly diagnosed ICH. The three groups involving different usage intensity levels were similar regarding the proportion of males and females, comorbidities, and comedications, but not antiarrhythmic agents (Table 1). The two statin solubility groups also exhibited similar proportions of males and females, comorbidities, and comedications, but not the comorbidity of ischemic stroke (Table 2). Moreover, 75.1% of the patients were moderate‐intensity statin users, and 59.4% were prescribed lipophilic statins.

**Table 1:** Baseline characteristics according to intensity of statin initiation; data presented as *n*, (%), *n* = 726

Characteristics	High (*n* = 92)	Moderate (*n *=* *545)	Low (*n *=* *89)	*P* a value
Age (Mean ± SD)	58.91 ± 12.70	60.72 ± 12.28	60.92 ± 12.48	0.4074
Male	52 (56.52)	300 (55.05)	53 (59.55)	0.7216
Charlson index				
0	40 (43.48)	234 (42.94)	31 (34.83)	0.5506
1	19 (20.65)	133 (24.40)	26 (29.21)	
≧2	33 (35.87)	178 (32.66)	32 (35.96)	
Comorbidities				
Hypertension	53 (57.61)	312 (57.25)	56 (62.92)	0.6013
Diabetes mellitus	25 (27.17)	152 (27.89)	27 (30.34)	0.8731
Hyperlipidemia	22 (23.91)	127 (23.30)	29 (32.58)	0.1668
Atrial fibrillation	3 (3.26)	19 (3.49)	2 (2.25)	0.8319
Coronary artery disease	15 (16.30)	89 (16.33)	15 (16.85)	0.9921
Ischemic stroke	18 (19.57)	95 (17.43)	13 (14.61)	0.6756
Co‐medication in previous 1 year				
Antiarrhythmic agents	7 (7.61)	75 (13.76)	18 (20.22)	0.0483^a^
Anticoagulant agents	7 (7.61)	55 (10.09)	9 (10.11)	0.7547
Antiplatelet agents	51 (55.43)	333 (61.10)	62 (69.66)	0.1376
Antidiabetic agents	40 (43.48)	265 (48.62)	40 (44.94)	0.5753
ACEi/ARB	81 (88.04)	507 (93.03)	80 (89.89)	0.1938
Beta blockers	80 (86.96)	494 (90.64)	82 (92.13)	0.4505
Calcium channel blockers	85 (92.39)	496 (91.01)	82 (92.13)	0.8719
Diuretics	72 (78.26)	428 (78.53)	71 (79.78)	0.9608
Digoxin	9 (9.78)	459 (15.78)	73 (17.98)	0.2526
Other dyslipidemic agents^b^	42 (43.48)	223 (40.92)	40 (44.94)	0.7256

ACEi, angiotensin‐converting enzyme inhibitor; ARB, angiotensin II receptor blocker.

High intensity: atorvastatin 40–80 mg, rosuvastatin 20–40 mg; Moderate intensity: atorvastatin 10–20 mg, rosuvastatin 5–10 mg, simvastatin 20–40 mg, pravastatin 40–80 mg, lovastatin 40 mg, fluvastatin XL 80 mg, fluvastatin 40 mg bid, pitavastatin 2–4 mg; Low intensity: simvastatin 10 mg, pravastatin 10–20 mg, lovastatin 20 mg, fluvastatin 20–40 mg, pitavastatin 1 mg.

a ANOVA for continuous variables; Chi‐squared test for categorical variables.

b Other dyslipidemic agents include cholestyramine, fibrates, and niacin.

**Table 2:** Baseline characteristics according to solubility of statin; data presented as *n*, (%), *n *=* *726

Characteristics	Lipophilic (*n *=* *431)	Hydrophilic (*n* = 295)	*P* a value
Age (Mean ± SD)	60.48 ± 12.58	60.57 ± 12.05	0.9279
Male	240 (55.68)	165 (55.93)	0.9474
Charlson index			
0	176 (40.84)	129 (43.73)	0.4637
1	103 (23.90)	75 (25.42)	
≧2	152 (35.27)	91 (30.85)	
Comorbidities			
Hypertension	247 (57.31)	174 (58.98)	0.6535
Diabetes mellitus	122 (28.31)	82 (27.80)	0.8807
Hyperlipidemia	109 (25.29)	69 (23.39)	0.5589
Atrial fibrillation	15 (3.48)	9 (3.05)	0.7506
Coronary artery disease	66 (15.31)	53 (17.97)	0.3430
Ischemic stroke	86 (19.95)	40 (13.56)	0.0255^a^
Co‐medication in previous 1 year			
Antiarrhythmic agents	62 (14.39)	38 (12.88)	0.5636
Anticoagulant agents	42 (9.74)	29 (9.83)	0.9695
Antiplatelet agents	277 (64.27)	169 (57.29)	0.0577
Antidiabetic agents	197 (45.71)	148 (50.17)	0.2371
ACEi/ARB	393 (91.18)	275 (93.22)	0.3201
Beta blockers	385 (89.33)	271 (91.86)	0.2553
Calcium channel blockers	390 (90.49)	273 (92.54)	0.3340
Diuretics	336 (77.96)	235 (79.66)	0.5824
Digoxin	64 (14.85)	47 (15.93)	0.6904
Other dyslipidemic agents^b^	172 (39.91)	131 (44.41)	0.2272

ACEi: angiotensin‐converting enzyme inhibitor; ARB: angiotensin II receptor blocker.

Hydrophilic solubility: pravastatin, rosuvastatin; Lipophilic solubility: atorvastatin, cerivastatin, fluvastatin, lovastatin, simvastatin.

a Student *t* test for continuous variables; Chi‐squared test for categorical variables.

b Other dyslipidemic agents include cholestyramine, fibrates, and niacin.

### Cumulative incidence and hazard ratio of the composite endpoints among the high‐, moderate‐, and low‐intensity groups during follow‐up

The endpoints were assessed in the three groups with varying statin intensities (high, moderate, and low; Table 3). During the follow‐up period, 135 (18.6%) patients developed one of the adverse outcomes of interest. Of these patients, 15 (16.3%) were high‐intensity statin users, 109 (20.0%) were moderate‐intensity users, and 11 (12.4%) were low‐intensity users (Table 3). Compared with the patients in the high‐intensity group, those in the low‐intensity group demonstrated no significant difference in the incidence of recurrent ICH (adjusted HR [aHR] = 0.66, 95% CI = 0.30–1.44), all‐cause mortality (aHR = 0.65, 95% CI = 0.28–1.55), ischemic stroke or TIA (aHR = 0.96, 95% CI = 0.67–1.38), or acute coronary events (aHR = 1.69, 95% CI = 0.17–16.68). Moreover, the patients in the moderate‐intensity group had no significant difference in the incidence of all‐cause mortality (aHR = 0.75, 95% CI = 0.38–1.48), recurrent ICH (aHR = 1.19, 95% CI = 0.69–2.05), acute coronary events (aHR = 2.15, 95% CI = 0.28–16.30), or ischemic stroke or TIA (aHR = 1.07, 95% CI = 0.81–1.42; Table 3).

**Table 3:** Multivariable‐adjusted relationships between the intensity (high, moderate, and low) of statin use and outcomes

Characteristics	High (*n* = 92)	Moderate (*n* = 545)	Low (*n* = 89)
Recurrent ICH, *n* (%)	15 (16.3)	109 (20.0)	11 (12.4)
Multivariable Adjusted OR (95% CI)	1.00 (–)	1.19 (0.69–2.05)	0.66 (0.30–1.44)
Death (all cause), *n* (%)	10 (10.9)	70 (12.8)	13 (14.6)
Multivariable Adjusted OR (95% CI)	1.00 (–)	0.75 (0.38–1.48)	0.65 (0.28–1.55)
Acute coronary event, *n* (%)	1 (1.1)	19 (3.5)	3 (3.4)
Multivariable Adjusted OR (95% CI)	1.00 (–)	2.15 (0.28–16.30)	1.69 (0.17–16.68)
Ischemic stroke/Transient ischemic attack, *n* (%)	58 (63.0)	380 (69.7)	66 (74.2)
Multivariable Adjusted OR (95% CI)	1.00 (–)	1.07 (0.81–1.42)	0.96 (0.67–1.38)

CI, confidence interval; ICH, intracerebral hemorrhage.

High intensity: atorvastatin 40–80 mg, rosuvastatin 20–40 mg; Moderate intensity: atorvastatin 10–20 mg, rosuvastatin 5–10 mg, simvastatin 20–40 mg, pravastatin 40–80 mg, lovastatin 40 mg, fluvastatin XL 80 mg, fluvastatin 40 mg bid, pitavastatin 2–4 mg; Low intensity: simvastatin 10 mg, pravastatin 10–20 mg, lovastatin 20 mg, fluvastatin 20–40 mg, pitavastatin 1 mg.

Adjusted variables include the following: age, sex, hypertension, diabetes mellitus, hyperlipidemia, atrial fibrillation, coronary artery disease, ischemic stroke, anticoagulant agents, antiplatelet agents, calcium channel blockers, diuretics, digoxin, antidiabetic agents, other dyslipidemic agents, and preuse of statin.

### Cumulative incidence and hazard ratio of the composite endpoints between hydrophilic and lipophilic groups during follow‐up

The endpoints were assessed in the two statin solubility groups (hydrophilic and lipophilic; Table 4). During the follow‐up period, 135 (18.6%) patients had one of the adverse study outcomes, and of these patients, 43 (14.6%) were users of hydrophilic statins and 92 (21.4%) were users of lipophilic statins. Compared with the patients using lipophilic statins, those using hydrophilic statins had a significantly lower incidence of recurrent ICH (aHR: 0.69, 95% CI: 0.48–0.99), but no significantly different incidence of all‐cause mortality (aHR = 1.15, 95% CI = 0.74–1.78), acute coronary events (aHR = 1.33, 95% CI = 0.57–3.15), or ischemic stroke or TIA (aHR = 1.09, 95% CI = 0.91–1.31; Table 4).

**Table 4:** Multivariable‐adjusted relationships between the solubility (hydrophilic and lipophilic) of statins and outcomes

Characteristics	Lipophilic (*n* = 431)	Hydrophilic (*n* = 295)
Recurrent ICH, *n* (%)	92 (21.4)	43 (14.6)
Multivariable Adjusted OR (95% CI)	1.00 (–)	0.69 (0.48–0.99)^a^
Death (all cause), *n* (%)	56 (13.0)	37 (12.5)
Multivariable Adjusted OR (95% CI)	1.00 (–)	1.15 (0.74–1.78)
Acute coronary event, *n* (%)	12 (2.8)	11 (3.7)
Multivariable adjusted OR (95% CI)	1.00 (–)	1.33 (0.57–3.15)
Ischemic stroke/Transient ischemic attack, *N* (%)	298 (69.1)	206 (69.8)
Multivariable adjusted OR (95% CI)	1.00 (–)	1.09 (0.91–1.31)

Hydrophilic solubility: pravastatin, rosuvastatin; Lipophilic solubility: atorvastatin, cerivastatin, fluvastatin, lovastatin, simvastatin.

Adjusted variables include the following: age, sex, hypertension, diabetes mellitus, hyperlipidemia, atrial fibrillation, coronary artery disease, ischemic stroke, anticoagulant agents, antiplatelet agents, calcium channel blockers, diuretics, digoxin, antidiabetic agents, other dyslipidemic agents, and preuse of statin.

ICH, intracerebral hemorrhage; CI, confidence interval.

a *P *<* *0.05.

**Figure 2:**
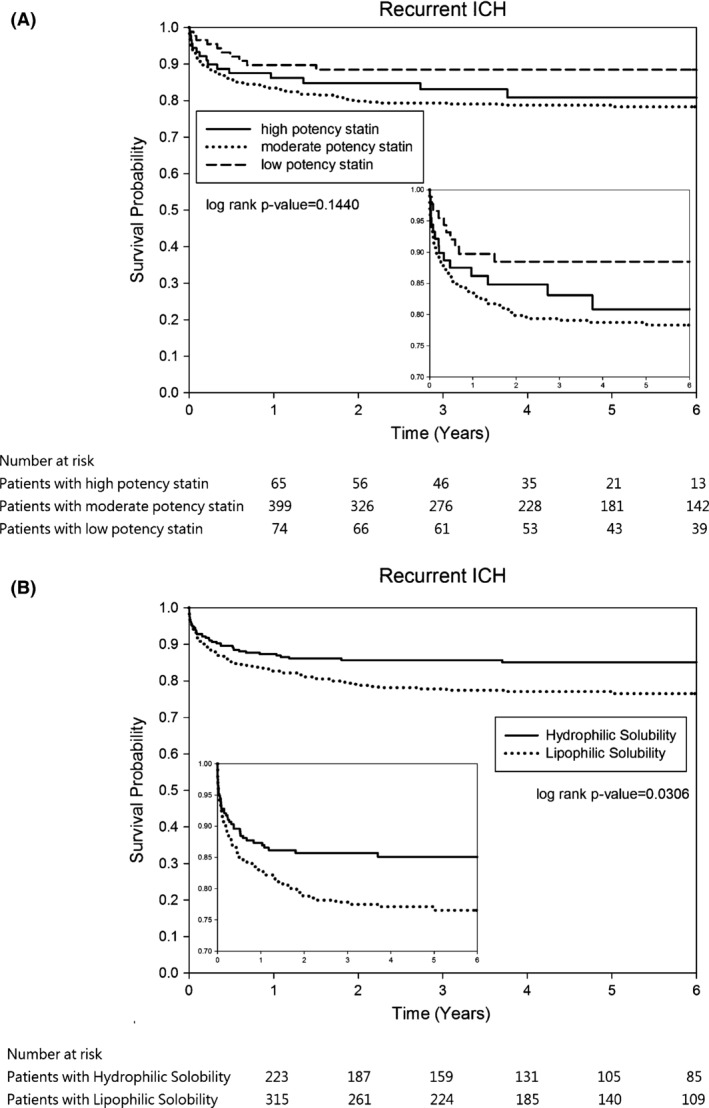
Kaplan–Meier survival curve for the probability of being event‐free at 6 years stratified by the intensity of statin therapy (A) and solubility of statin therapy (B).

Figure 2 illustrates a Kaplan–Meier curve representing the probability of an event occurring during the follow‐up period in the three statin‐intensity groups and two solubility groups. Although we observed no significant differences in the recurrence of ICH (*P* = 0.1440) among the three statin intensity groups, we determined a significant difference (*P* = 0.0306) in the recurrence of ICH between the statin solubility groups.

## Discussion

By using Taiwan's nationwide NHI database, we determined that newly diagnosed ICH patients treated with hydrophilic statins had a lower risk of recurrent ICH during the follow‐up period than did those treated with lipophilic statins. We observed no significant relationship between the intensity of statin use and recurrent ICH or secondary outcomes.

The benefits of HMG‐CoA reductase inhibitors (statins) for the primary and secondary prevention of cardiac and cerebrovascular disease are adequately established (Amarenco et al. 2006; Gaspardone and Arca 2007; Ward et al. 2007). However, a previous trial, Stroke Prevention by Aggressive Reduction in Cholesterol Levels (SPARCL), identified a minor increase in the risk of ICH (HR: 1.66; 95% CI: 1.08–2.55) associated with high‐dose statin use (Goldstein et al. 2008). Although another meta‐analysis found no association between statin use or low‐density cholesterol levels and ICH, statin therapy among patients at high risk for hemorrhage because of prior ICH still remains a concern. Furthermore, ICH survivors commonly have comorbid cardiovascular risk factors that would otherwise warrant cholesterol‐lowering medication; achieving a balance between the risk of ICH and ischemic vascular diseases for statin therapy in this population is considerably challenging.

In addition to cholesterol reduction (Takemoto and Liao 2001; Reiss and Wirkowski 2007), statins have pleiotropic effects, and some of them have been suggested to be possible mechanisms underlying increased ICH risks (Meier et al. 2009). For example, evidence shows that statins have antithrombotic (Szapary et al. 2004) and fibrinolytic (Aarons et al. 2007) effects in addition to enhancing the activity of other fibrinolytic agents (Undas et al. 2006). In a previous study, an association between increasing doses of statin and the risk of ICH was observed among European patients with acute ischemic stroke after intravenous thrombolysis (Scheitz et al. 2014). Another animal study revealed that high‐dose statin use exerted adverse effects on function and tissue recovery and that statin‐induced adverse effects may be dose‐related (Yang et al. 2013a,b). The mechanisms underlying the absence of a therapeutic effect of high‐dose statin use in this ICH model remain unknown. Historically, an elevated dosage of statins was believed to reduce the level of cholesterol linked with an increased rate of hemorrhagic stroke (Tirschwell et al. 2004; Goldstein et al. 2008). In addition, reports have suggested that the more lipid‐soluble a statin is, the greater is its propensity to cross the blood–brain barrier and affect the central nervous system and vessels in the brain (Goldstein et al. 2004). Therefore, potency and lipophilic solubility are expected to correlate with both the protective and adverse effects of statin usage. In addition, previous studies have compared statin users with nonstatin users (Hackam et al. 2012; Mckinney and Kostis 2012; Laloux 2013; Liu et al. 2013; Lei et al. 2014; Asberg and Eriksson 2015), and a recent nationwide observational study demonstrated that statin therapy was associated with a lower risk of ICH compared with nonstatin therapy (Asberg and Eriksson 2015). Most physicians in Taiwan comply with insurance regulations and prescribe statins to patients exhibiting related risk factors and high lipid profiles, satisfying the criteria of the NHI guidelines. Regarding comparability, we examined the relationship between the characteristics of statin use and the outcomes in post‐ICH patients, and we determined that the risks of developing recurrent ICH between the low‐ or moderate‐intensity users and the high‐intensity users were not significantly different. However, we identified hydrophilic statin users to be at significantly lower risk of developing recurrent ICH compared with lipophilic statin users. Therefore, compared with the intensity, which included dosage and potency, solubility may have been more critical to the safety of statin use. The findings of our study may serve as a reference for selecting statin regimens. Prospective randomized clinical studies are required to confirm these findings.

This study had several limitations. First, the diagnoses of hemorrhagic stroke in the NHIRD were not validated, and a misclassification of events might have occurred. To limit this possibility, we recruited only participants who were admitted to hospitals with a first diagnosis of ICH and subjected to a CT scan during the admission. These criteria were also applied to the outcome of recurrent ICH. Second, we could not control for out‐of‐pocket purchases and adherence to prescribed statin medication regimens, which could have resulted in a misclassification of exposure. However, this misclassification would have been unlikely because all statin products are reimbursable by the NHI program in Taiwan and because the problem of adherence to medication regimens has similar effects on each group. Third, serum cholesterol levels could not be identified in the claims records and were thus unavailable in our study. Hence, we could not evaluate the association between serum cholesterol levels and the risk of ICH. Nevertheless, data from the SPARCL trial (Goldstein et al. 2008) revealed no relationship between on‐treatment, low‐density lipoprotein cholesterol levels and the risk of hemorrhagic stroke in statin‐treated patients. Fourth, ICH is a heterogeneous entity with different locations, origins, and recurrence risks. In ICH survivors, the rate of ICH recurrence is different; this recurrence depends on various factors such as the type of bleeding, ex.: arterio‐venous malformation (AVM), cerebral aneurysm, and cerebral amyloid angiopathy (Viswanathan et al. 2006). In this study, we identified only two patients with AVM and three with cerebral amyloid angiopathy. The result was similar after these patients were excluded. However, we did not include detailed information for determining which location of cerebral bleeding might be influenced by statin, and our results might be carefully applied to a specific subtype of ICH patients (e.g., patients with lobar ICH). Fifth, although statistical adjustment was performed using multivariable models, residual, unmeasured confounders (e.g., cigarette smoking and excessive alcohol consumption) remain a concern. Finally, because our study was based on a homogeneous Asian population, the generalizability of our findings to other racial groups may be limited.

## Conclusion

This 10‐year nationwide follow‐up cohort study of newly diagnosed ICH patients receiving statin therapy demonstrated that users of hydrophilic statins had a significantly lower risk of recurrent ICH than did users of lipophilic statins. Additionally, the intensity of statin use had no significant relationship with recurrent ICH or other secondary adverse outcomes. Additional prospective randomized clinical trials are required to confirm the favorable effects of hydrophilic statin therapy in ICH patients.

## Conflict of Interest

None declared.
